# Line-Field Confocal Optical Coherence Tomography: A New Tool for the Differentiation between Nevi and Melanomas?

**DOI:** 10.3390/cancers14051140

**Published:** 2022-02-23

**Authors:** Sandra Schuh, Cristel Ruini, Maria Katharina Elisabeth Perwein, Fabia Daxenberger, Charlotte Gust, Elke Christina Sattler, Julia Welzel

**Affiliations:** 1 Department of Dermatology and Allergology, University Hospital, 86179 Augsburg, Germany; maria.perwein@web.de; 2 Department of Dermatology and Allergy, University Hospital, LMU Munich, 80337 Munich, Germany; cristel.ruini@med.uni-muenchen.de (C.R.); fabia.daxenberger@gmail.com (F.D.); charlotte.gust@t-online.de (C.G.); elke.sattler@med.uni-muenchen.de (E.C.S.)

**Keywords:** melanoma, nevus, skin cancer, line-field confocal optical coherence tomography, optical coherence tomography, reflectance confocal microscopy, skin imaging, non-invasive diagnostics in dermatology

## Abstract

**Simple Summary:**

Typical benign nevi and advanced melanomas can be easily discriminated, but there are still some melanocytic lesions where even experts are not sure about the correct diagnosis and degree of malignity. The high penetration depth of optical coherence tomography (OCT) allows an assessment of tumor thickness of the lesion precisely, but without cellular resolution the differentiation of melanocytic lesions remains difficult. On the other hand, reflectance confocal microscopy (RCM) allows for very good morphological identification of either a nevus or a melanoma, but cannot show the infiltration depth of the lesion because of its low penetration depth. Since the new device of line-field confocal optical coherence tomography (LC-OCT) technically closes the gap between these other two devices, in this study, we wanted to examine if it is possible to differentiate between nevi and melanomas with LC-OCT, and which criteria are the most important for it.

**Abstract:**

Until now, the clinical differentiation between a nevus and a melanoma is still challenging in some cases. Line-field confocal optical coherence tomography (LC-OCT) is a new tool with the aim to change that. The aim of the study was to evaluate LC-OCT for the discrimination between nevi and melanomas. A total of 84 melanocytic lesions were examined with LC-OCT and 36 were also imaged with RCM. The observers recorded the diagnoses, and the presence or absence of the 18 most common imaging parameters for melanocytic lesions, nevi, and melanomas in the LC-OCT images. Their confidence in diagnosis and the image quality of LC-OCT and RCM were evaluated. The most useful criteria, the sensitivity and specificity of LC-OCT vs. RCM vs. histology, to differentiate a (dysplastic) nevus from a melanoma were analyzed. Good image quality correlated with better diagnostic performance (Spearman correlation: 0.4). LC-OCT had a 93% sensitivity and 100% specificity compared to RCM (93% sensitivity, 95% specificity) for diagnosing a melanoma (vs. all types of nevi). No difference in performance between RCM and LC-OCT was observed (McNemar’s *p* value = 1). Both devices falsely diagnosed dysplastic nevi as non-dysplastic (43% sensitivity for dysplastic nevus diagnosis). The most significant criteria for diagnosing a melanoma with LC-OCT were irregular honeycombed patterns (92% occurrence rate; 31.7 odds ratio (OR)), the presence of pagetoid spread (89% occurrence rate; 23.6 OR) and the absence of dermal nests (23% occurrence rate, 0.02 OR). In conclusion LC-OCT is useful for the discrimination between melanomas and nevi.

## 1. Introduction

Optical coherence tomography (OCT) and reflectance confocal microscopy (RCM) already play important roles in routine non-invasive skin cancer diagnosis [1]. OCT is well established for non-melanoma skin cancer (NMSC), whereas RCM is most commonly used for pigmented lesions [2,3,4]. With OCT it is possible to create vertical images until mid to deep dermal level, to reconstruct 3D images, measure tumor thickness, monitor topically treated lesions over the course of time, and even to distinguish BCC subtypes, but lacks cellular resolution [2,3]. This is why melanocytic lesions cannot be visualized very well with OCT. With the high resolution of RCM, pigmented tumors can be examined horizontally in cellular detail. Since the penetration depth of RCM is limited, it is only possible to measure from the stratum corneum to the stratum papillare of the dermis [4,5]. 

The new line-field confocal optical coherence tomography (LC-OCT) method offers the combination of the advantages of both devices—a high penetration depth like OCT and a similar high resolution like RCM, together with the visualization of images in horizontal and vertical views [6,7,8,9,10,11].

Therefore, LC-OCT was used in this study to investigate melanocytic lesions and to find out the most common and most useful parameters for diagnosis, especially for the differentiation between (dysplastic) nevi and melanomas. The improved distinction between nevus and melanoma with LC-OCT allows a fast, in vivo, and non-invasive diagnosis. If the lesion can be categorized as a nevus immediately with LC-OCT, no excision is necessary. In addition to the benefit to the patient that no invasive procedure is needed, this new technique will help to avoid unnecessary surgeries, leading to lower healthy insurance costs, as well as to offering more free capacities for other necessary melanoma surgeries. This study assesses the influence of image quality on diagnostic performance with LC-OCT and RCM and determines the potential of LC-OCT (vs. RCM vs. histology) to identify a melanoma vs. a nevus reliably and accurately. To our knowledge, this is the first work about melanocytic lesions, especially concerning the differentiation of nevi and melanomas with the new LC-OCT device.

## 2. Materials and Methods

### 2.1. LC-OCT

The LC-OCT device used in our study is based on a time-domain-OCT (TD-OCT), which takes several A-scans from the skin’s surface down to a maximal depth of 500 µm for the acquisition of B-scans, while constantly refocusing. It has an axial resolution of 1.1 µm and a lateral resolution of 1.3 µm. The LC-OCT technique consists of a two-beam interference microscope, with a laser source of 800 nm wavelength with a continuous spectrum and a line camera as a photodetector. The laser classification is 1 M according to EN 60825-1. Like in conventional OCT, the images are depicted in grey scale. The CE-marked LC-OCT deepLive^TM^ (DAMAE Medical, Paris, France) is a mobile central unit with a monitor and a handheld probe. To generate high quality images and reduce the optical index between the glass plate of the probe and skin layers, paraffin oil was used. The device is non-invasive, painless, and is applied without pressure. LC-OCT has three available modes to create images in real time: vertical (en-coupe) sections, as in OCT and histology, horizontal (en-face) images as in RCM, and 3D images. The field of view for both the vertical and horizontal sections is 1.2 mm × 0.5 mm. The 3D images can be taken as horizontal stacks from the top of the skin with steps of 1 µm. Short videos can also be acquired with up to 26, 16, or 8 frames/s (basic, high definition, ultra-high definition). Moreover, a dermoscopic image (resolution 5 µm, field of view 2.5 mm) is taken simultaneously, which makes it possible to navigate in the lesion and choose the correct position. Captured images can be exported and saved globally or as a single patient/lesion file in TIFF, DICOM, or JPEG formats. The additional program 3DSlicer (The Slicer Community, Open-Source Software) allows the reconstruction of 3D cubes of the horizontal LC-OCT stacks [9,10].

### 2.2. RCM and OCT

In our study we used commercially distributed RCM devices (VivaScope^®^ 1500 and VivaScope^®^ 1500/3000 Combo, Mavig GmbH, Munich, Germany), which have a penetration depth of 300 µm. Both have a lateral resolution of 1 µm and an axial resolution between 3–5 µm. The light source is an 830 nm diode laser. The 1500 device has an integrated dermoscopic camera for navigation, creates image sizes of 500 µm × 500 µm, and builds mosaics of single greyscale pictures up to 8 mm × 8 mm (VivaBlock^®^). The other mode, which we used in the study for the comparison with LC-OCT, is the VivaStack^®^, where single images can be taken from the skin’s surface to the stratum papillare in several steps. The conventional OCT used in this study is the commercially available, handheld-based OCT device VivoSight^®^ (Michelson Diagnostics Ltd., Maidstone, Kent, UK). We additionally measured 30 of all the melanocytic lesions with conventional OCT for comparative reasons. The OCT acquires images of 6 mm × 6 mm with 1.5 mm detection depth. It has a lateral resolution of 7.5 µm and an axial resolution of 10 µm, and contains a laser source of 1305 nm. With the addition of dynamic OCT, blood flow and blood vessels can be visualized. Further details on the devices are described elsewhere [2,12].

### 2.3. Patients

Patients with suspicious melanocytic lesions or nevi that were planned for excision were enrolled in the study at the Department of Dermatology and Allergology at the University Hospital of Augsburg and at the University Hospital of the Ludwig Maximilian University, Munich in Germany between November 2019 and January 2021. The study was approved by the local ethics committee (No. 17-699) and conducted according to the principles of the Declaration of Helsinki and international guidelines concerning human studies. Written informed consent was obtained from all patients prior to inclusion into the study.

### 2.4. Measurements

A clinical examination was done to identify the lesion as suspicious for a melanoma or a (dysplastic) nevus prior to excision. Clinical and dermoscopic images of each lesion were acquired using Fotofinder^®^ (FotoFinder Systems GmbH, Bad Birnbach, Germany), DermoGenius 2^®^ (DermoScan GmbH, Regensburg, Germany), ILLUCO IDS-1100 (DermoScan GmbH, Regensburg, Germany), or an iPhone 12 Pro Camera (Apple Inc., Cupertino, CA, USA). After dermoscopy, the lesions were scanned with LC-OCT (deepLive^TM^, DAMAE Medical, Paris, France) in horizontal, vertical, and 3D. After that, OCT images (vertical and dynamic en-face) as well as RCM pictures were taken (VivaStacks^®^ and VivaBlocks^®^). Horizontal LC-OCT and RCM images were taken at the level of the stratum corneum, the epidermis, the dermo-epidermal junction (DEJ), and the papillary dermis. For measurements with RCM and LC-OCT, a few drops of paraffin oil were used before the scan. OCT examinations did not require preparation of the skin. After the examination, a biopsy, shave, or excision of the lesion was taken and sent for histological analysis. All lesions were compared with standard histology in haematoxylin/eosin. 

After all images had been reviewed, the observers graded LC-OCT and RCM image quality during a consensus meeting with semi-quantitative scores as poor (3), acceptable (2), good (1), or excellent (0), and the confidence level from low (3), medium (2), high (1), to very high (0). After histology, the final diagnosis was noted. Both centers are regular users of OCT, RCM, and LC-OCT. Both centers had at least 3 months of practical experience and training with LC-OCT before the study. 

In addition to the well-known clinical and dermoscopic diagnostic criteria for melanocytic lesions, we used the following patterns for the different methods: LC-OCT criteria in horizontal and vertical view (see Tables S1 and S2) and RCM parameters (see Tables S3 and S4). In 30 cases, conventional OCT images were also recorded for demonstration purposes. Since RCM is superior to OCT in diagnosing melanocytic lesions and OCT cannot show the cellular details of melanomas and nevi, OCT criteria were not evaluated and OCT was not included in the systematic study [13,14].

### 2.5. Statistical Analysis 

For the collection of data, Microsoft^®^ Office Excel^®^ for Mac 2021 was used. Statistical analysis was performed with R version 4.0.0 (R Foundation for Statistical Computing; Vienna, Austria). The accuracy, specificity, sensitivity of each technique, PPV (percentage of positive diagnoses that were correct), and NPV (percentage of negative diagnoses that were correct) were calculated. The specificity and sensitivity of LC-OCT for the diagnosis of a melanoma were compared with the specificity and sensitivity of RCM, and then both devices with histology using McNemar’s test, which considers the paired nature of the data. Furthermore, the investigators’ responses concerning the recognized parameters for melanocytic lesions, the diagnoses, the image quality, and confidence level were noted. For the intra-method correlation between image quality and confidence level, Spearman’s correlation coefficients (r) were calculated. A *p*-value < 0.05 was considered as statistically significant. When the data failed the normality test, a Wilcoxon test for paired tests was used to compare image quality and confidence level between LC-OCT and RCM. We also performed univariate and multivariate logistic regression analyses for the criteria that were more useful in discriminating a melanoma from a nevus (dysplastic or not), and used a backward elimination approach. 

## 3. Results

### 3.1. Study Population

A total of 75 patients with 84 melanocytic lesions were evaluated with LC-OCT before excision. A total of 36 of the 84 lesions were also measured with RCM. Five patients had two lesions, one patient had five lesions, and the others had one pigmented lesion. The mean age of the patients was 51 years. Lesions were mainly located on the trunk (57.1%), limb (25%), and the head (17.9%). Histology was available for all 84 lesions. Histology identified 42 (50%) of 84 lesions as banal nevi, 13 (15.5%) as dysplastic nevi and 28 (33.3%) as melanomas. A total of 65.5% of the lesions were histologically diagnosed as nevi (dysplastic or not). Furthermore, 82.1% of the lesions were completely excised, 16.7% were biopsied, and 1.2% were shaved. One lesion was histologically diagnosed as a pigmented actinic keratosis and was therefore excluded for further evaluation. In terms of invasion levels, nine melanomas were in situ melanomas (32%), and nine invasive melanomas were up to 1 mm thick (32%). A total of seven melanomas had a tumor thickness between 1 and 2 mm (25%), two melanomas were between 2 and 4 mm thick (7%), and one melanoma was thicker than 4 mm (4%). Regarding the subtype, 17 melanomas were classified as superficial spreading melanomas (61%), 2 were nodular (7%), 2 were acrolentiginous (7%), and 7 lentigo maligna (3) or invasive lentigo maligna melanomas (4) (25%). TNM classifications for the melanomas were T0 (9), IA (9), IB (7), IIA (1), IIB (1), and IIIC (1). All melanomas with a tumor thickness up to 2 mm were excised with a safety margin of 1 cm, while melanomas with a tumor thickness ≥ 2mm received excision with a safety margin of 2 cm. In patients with a melanoma with a tumor thickness > 1 mm, a sentinel lymph node biopsy (or in high-risk cases with a tumor thickness > 0.75 mm) was conducted.

### 3.2. LC-OCT and RCM Image Quality and Confidence Level 

**Figure 1:**
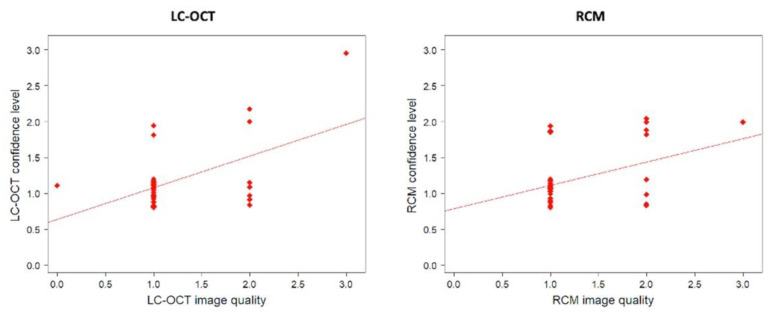
Correlation between confidence level and image quality for LC-OCT and RCM. There was a statistically significant positive correlation between LC-OCT confidence levels and image quality for LC-OCT (*r*_s_(36) = 0.40; *p* = 0.02), as well as for RCM (*r*_s_(36) = 0.43; *p* = 0.008).

A total of 1 out of 36 lesions imaged with RCM and 4 from 84 lesions imaged with LC-OCT were classified as having poor image quality. The diagnostic confidence level depended on image quality for LC-OCT and RCM (Table 1, Figure 1). 

Lower image quality led to a poorer confidence level (Table 2). We found no difference between RCM and LC-OCT in terms of average image quality or average diagnostic confidence level (Table 2). A total of 77.8% of the lesions had good LC-OCT image quality (scored 0 or 1), while 72.2% had good RCM image quality. The average quality was evaluated as 1.2 for LC-OCT vs. 1.3. for RCM. However, the image quality (*p* = 0.49) and the confidence level (*p* = 0.40) were not significantly different between LC-OCT and RCM (*p* = 0.49). In total, 86.1% of the lesions had a high LC-OCT confidence level (scored 0 or 1), while 77.8% had a high RCM confidence level. The average confidence level was calculated as 1.2 for LC-OCT vs. 1.2 for RCM. 

### 3.3. LC-OCT and RCM Performance for Diagnosing a Melanoma vs. a Nevus

Table 3 shows LC-OCT performances for diagnosing a melanoma vs. a nevus (dysplastic or not), and Table 4 for diagnosing a melanoma vs. a nevus vs. a dysplastic nevus for all 84 lesions. 

**Figure 2:**
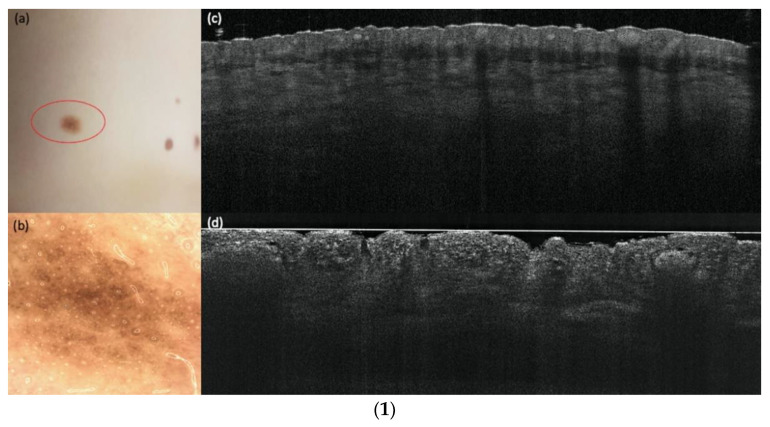

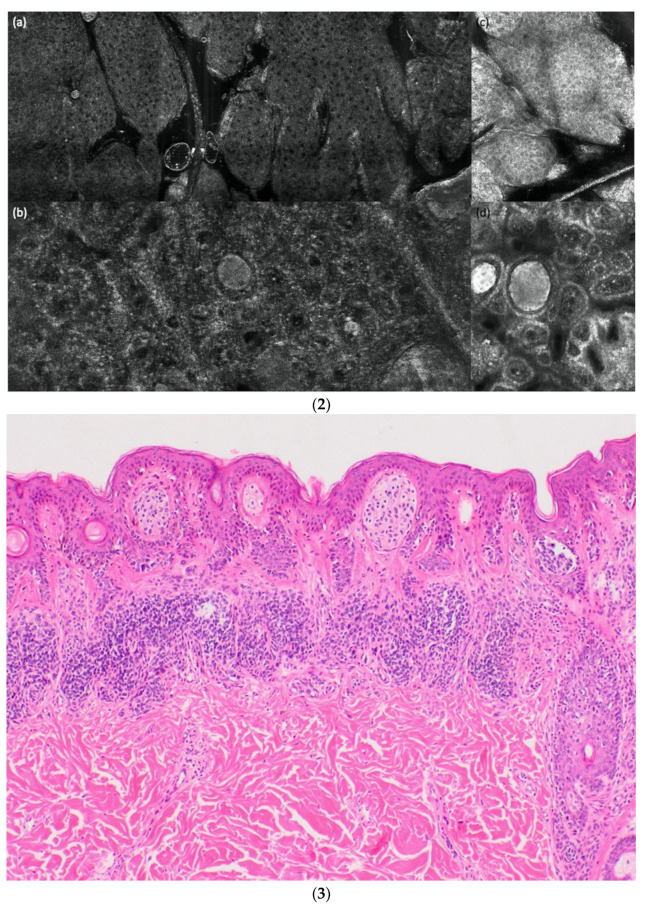
(**1**) A compound nevus in comparison with vertical LC-OCT and OCT. A compound nevus on the left lumbal trunk. (**a**) Clinical, (**b**) dermoscopical, and (**c**) optical coherence tomography (OCT; 6 mm × 2 mm) images of a representative nevus of the study. (**d**) In vertical line-field optical coherence tomography (LC-OCT; 1.2 mm × 0.5 mm) images junctional and dermal nests, a well-defined dermo-epidermal junction (DEJ), and a papillomatous surface can be seen. (**2**). A compound nevus in comparison with horizontal LC-OCT and RCM. The same compound nevus is depicted in horizontal LC-OCT (1.2 mm × 0.5 mm) with a regular honeycomb pattern (**a**), with nests in the upper dermis and regular papillae (**b**). Reflectance confocal microscopy (RCM; 500 µm × 500 µm) shows the same features. A regular honeycomb pattern (**c**) and junctional nests, as well as regular papillae, but just a little bit brighter (**d**). (**3**). A compound nevus in histology. The histology shows the same compound nevus as in (**1**) and (**2**) with 4× magnification.

**Figure 3:**
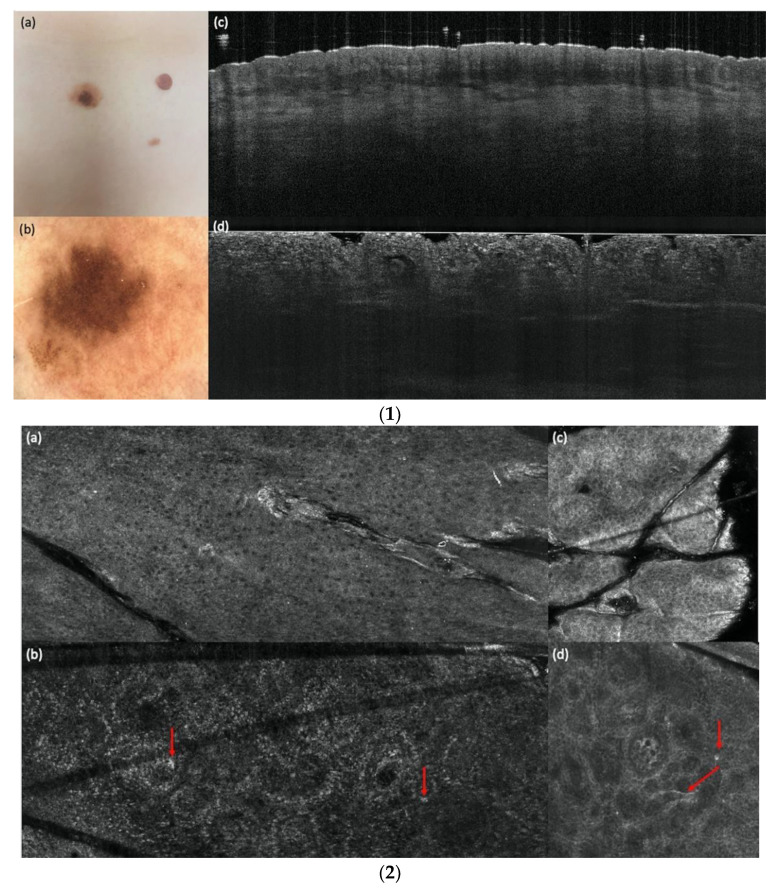

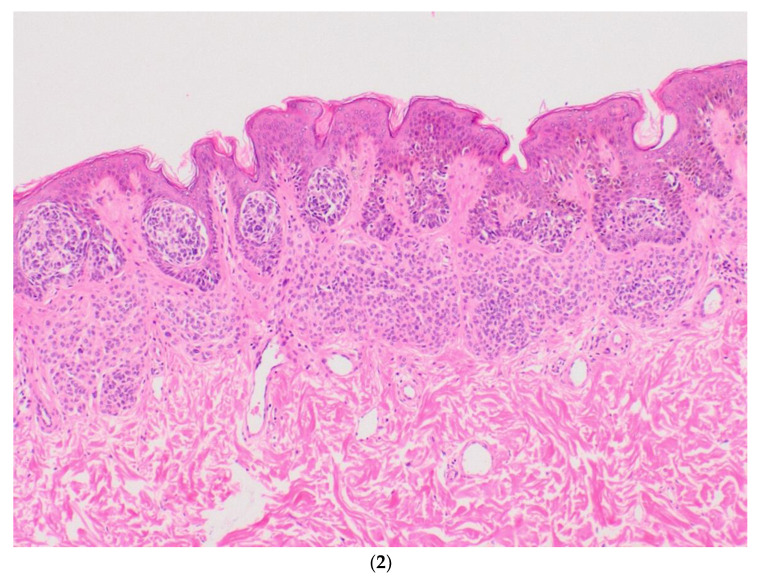
(**1**). A dysplastic compound nevus in comparison with vertical LC-OCT and OCT. A dysplastic nevus on the left lower trunk. (**a**) Clinical, (**b**) dermoscopical, and (**c**) optical coherence tomography (OCT; 6 mm × 2 mm) images of a representative dysplastic nevus of the study. (**d**) In vertical line-field optical coherence tomography (LC-OCT; 1.2 mm × 0.5 mm) images, junctional and dermal nests, a well-defined dermo-epidermal junction (DEJ), but also a few bright atypical cells are visible. (**2**) A dysplastic compound nevus in comparison with horizontal LC-OCT and RCM. The same dysplastic compound nevus is depicted in horizontal LC-OCT (1.2 mm × 0.5 mm) with a regular honeycomb pattern (**a**), with nests in the upper dermis, less regular papillae, and a few bright atypical cells (red arrows) (**b**). Reflectance confocal microscopy (RCM; 500 µm × 500 µm) shows the same features. A regular honeycomb pattern (**c**) and junctional nests, less regular papillae, and also a few bright atypical cells (red arrows) (**d**). (**3**) A dysplastic compound nevus in histology. The histology shows the same compound nevus as in (**1**) and (**2**) with 4× magnification.

**Figure 4:**
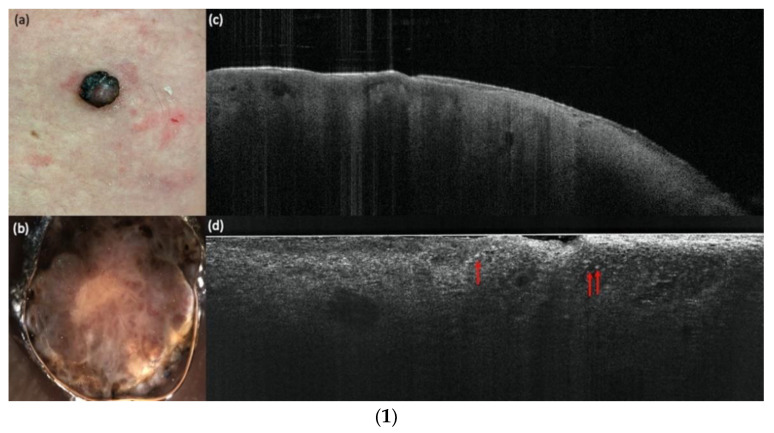

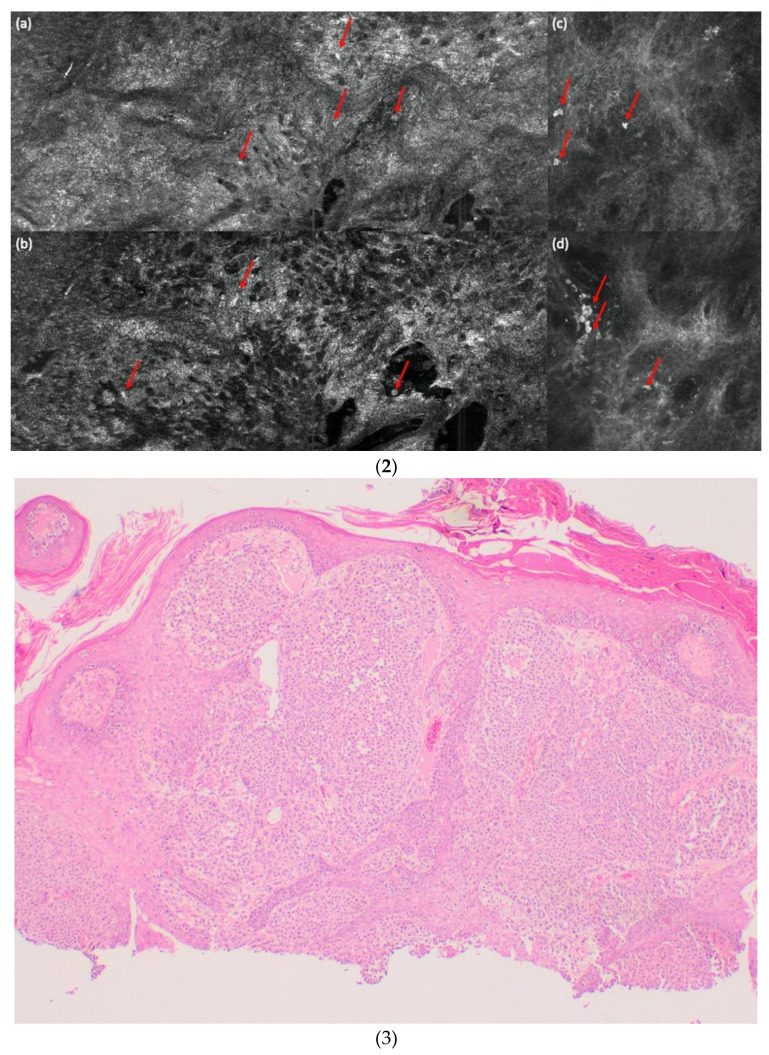
(**1**) A melanoma in comparison with vertical LC-OCT and OCT. An ulcerated nodular melanoma on the upper right leg. (**a**) Clinical, (**b**) dermoscopical, and (**c**) optical coherence tomography (OCT; 6 mm × 2 mm) images of a representative melanoma of the study. (**d**) In vertical line-field optical coherence tomography (LC-OCT; 1.2 mm × 0.5 mm) images, bright atypical melanocytic cells (red arrows) and a disturbed dermo-epidermal junction (DEJ) are visible. No dermal nests can be seen. (**2**) A melanoma in comparison with horizontal LC-OCT and RCM. The same melanoma is depicted in horizontal LC-OCT (1.2 mm × 0.5 mm) with an irregular honeycomb pattern with single atypical cells (red arrows) (**a**), and a pagetoid spread with atypical melanocytes (red arrows) (**b**). There were no dermal nests, a more chaotic structure, and no edged papillae visible. Reflectance confocal microscopy (RCM; 500 µm × 500 µm) shows the same features. An irregular honeycomb pattern with single atypical cells, just brighter (red arrows), (**c**) and a lighter pagetoid spread with atypical melanocytes (red arrows) (**d**). There were also no dermal nests, a more chaotic structure, and no edged papillae visible. (**3**) A melanoma in histology. The histology shows the same ulcerated nodular melanoma as in (**1**) and (**2**) with a tumor thickness of 4.8 mm and with 4× magnification.

Figure 2 shows an example of a compound nevus, Figure 3 a dysplastic nevus and Figure 4 a melanoma with all three devices compared to histology. The case diagnosed as “other” (pigmented actinic keratosis) in histology was dropped for the evaluation. 

The accuracy of all LC-OCT performances for diagnosing a melanoma vs. nevus was 97.6%, the sensitivity was 92.9%, and the specificity was 100% (see Table 5). The specificity and sensitivity of LC-OCT for diagnosing a melanoma vs. a nevus were compared with the specificity and sensitivity of histology using McNemar’s test (*p* = 0.48). Two melanomas were falsely negative and were diagnosed as nevi with LC-OCT (for the analysis, see Table S5). 

Table 6, Table 7 and Table 8 illustrate the subgroup of 36 lesions that were imaged with LC-OCT and RCM. The accuracy of all performances with LC-OCT was 97.1% vs. 94.3% with RCM, the sensitivity for both was 92.9%, and the specificity with LC-OCT was 100% vs. 95.2% with RCM. The specificity and sensitivity of both devices for diagnosing a melanoma vs. a nevus were compared with the specificity and sensitivity of histology and with each other using McNemar’s test (for all *p* = 1). One false negative was found, which was the same lesion for LC-OCT and RCM, and which had a bad image quality score in LC-OCT (3) and in RCM (2). 

The most significant criteria for diagnosing a melanoma with LC-OCT were irregular honeycombed pattern (92% occurrence rate; 31.7 OR), presence of pagetoid spread (89 % occurrence rate; 23.6 OR) and absence of dermal nests (23 % occurrence rate, 0.02 OR) as seen in Table 9.

## 4. Discussion

Due to the high resolution and good penetration depth of LC-OCT, it is finally possible to overcome the gap between OCT and RCM. The vertical view is similar to OCT, and visualizes single cells, making the comparison with histology very intuitive. Monnier et al. already proved that LC-OCT can discriminate different skin levels and keratinocytes in healthy skin acquisitions [15,16,17]. Recent studies show that with single cell display, even BCC subtypes can be discriminated in vertical LC-OCT [18]. The disadvantage of LC-OCT is the lower penetration depth compared to OCT, so it is possible that deeper tumor parts may be missed [19,20]. Due to the presentation of single cells, differential diagnoses such as sebaceous hyperplasia, actinic keratoses, and squamous cell carcinomas could be differentiated, and the proliferation degree of actinic keratoses showed a 75% concordance between LC-OCT and histology [21,22,23,24]. 

This high single cell resolution is the reason why we assumed that even melanocytic lesions can be evaluated with LC-OCT. Regarding resolution, the horizontal LC-OCT images were very similar to RCM, which is the reference standard. With RCM it has already been shown that nevi can be discriminated from melanomas, even if there are sometimes possible false-negative and false-positive cases of melanomas [25]. Moreover, ex vivo RCM has also been successfully used for the differentiation and diagnosis of melanocytic lesions, although not all typical in vivo features could be detected since it offers a vertical view instead of horizontal as in in vivo RCM [26]. Hartmann et al. stated that ex vivo RCM might also be useful in the measurement of tumor thickness, and therefore might be of help for the presurgical definition of correct margins [27]. LC-OCT is similar to a fusion of ex vivo and in vivo RCM. It provides the vertical view of ex vivo RCM and the horizontal view of in vivo RCM on melanocytic lesions. Therefore, more information on pigmented lesions can be gained, and more typical features of both techniques can lead the clinician to the correct diagnosis. In our study, we found that image quality was responsible for the diagnostic confidence level for LC-OCT and RCM. In general, reduced image quality is mainly associated with ulceration, crusting, or image taking experience. Nevertheless, it is not recommended to measure an ulcerated or crusted pigmented lesion with non-invasive devices, and in such cases a biopsy is needed. Clearly, the diagnostic confidence for each lesion depends not only on the quality of the LC-OCT image or the difficulty of the lesion, but also on the experience of the observer. Our clinicians were all similarly experienced, and therefore we did not evaluate observer variability. Hence, further studies with clinicians of different experiences with LC-OCT need to be performed. We also conclude that for the interpretation and analysis of LC-OCT images, tele-consulting might be useful for LC-OCT beginners and for discussing difficult cases. Furthermore, the diagnostic performance of LC-OCT for melanomas (vs. all nevi) has the same sensitivity and a better specificity compared to RCM. We only had two false negative cases with LC-OCT, where two melanomas were diagnosed as nevi. We reviewed the cases, and in the first case—a nevus-associated melanoma—we detected in the integrated dermoscopic view that only the dysplastic nevus part had been imaged. Thus, it is very relevant to ensure appropriate coverage of the whole lesion, or if the lesion is too big, that more images are taken. The second case—a big in situ superficial spreading melanoma—was of a bad image quality, more in LC-OCT (score 3) than in RCM (score 2), because of an air bubble due to technical issues at that time. In big-sized lesions, multiple measurements from different parts should have been taken. Due to the lesion’s large size, a biopsy or surgery would have been performed anyway. Moreover, the diagnosis of a nevus vs. a dysplastic nevus is less accurate for both techniques. This aspect needs to be reconsidered, since one limitation of our study is that the number of dysplastic nevi in the subgroup of both devices was quite small (*n* = 3) vs. nevi (*n* = 19). A larger study for the evaluation of nevi vs. dysplastic nevi is required. One should keep in mind that histology can also be erroneous, especially in biopsies, because the entire lesion cannot be assessed here. Since the vast majority of the lesions were completely excised and serial sections including immunohistologies are standard in melanomas, we assume that this possible error is negligible. 

Recently Lenoir et al. published a few LC-OCT criteria for benign dermal melanocytic proliferations such as wave pattern [28]. In our study, we evaluated the most significant criteria for diagnosing a melanoma vs. nevus with LC-OCT (in comparison with RCM). We found that an irregular honeycombed pattern, the presence of pagetoid spread, and the absence of dermal nests are the most important criteria to discriminate a melanoma from a nevus in LC-OCT. These findings are very similar to studies with RCM [29,30,31]. We were surprised that no criteria related to the DEJ could be detected here, but this can be explained by the fact that many superficial spreading melanomas have a well-defined DEJ, which were the majority of the melanomas in our study. In the future, a study about thin versus thick melanomas should be conducted, since Rudnicka et al. showed in RCM that there might be different key criteria [32].

## 5. Conclusions

In conclusion, our first study with the new LC-OCT device on melanocytic lesions showed that the discrimination between melanomas and nevi is possible. The improved distinction between nevi and melanomas with LC-OCT will lead to an immediate in vivo and non-invasive diagnosis, spare unnecessary surgeries if the lesion is diagnosed as a nevus, and therefore reduce health insurance costs and free capacities for other necessary melanoma surgeries.

## Figures and Tables

**Table 1:** Influence of image quality on the LC-OCT and RCM confidence level. LC-OCT and RCM image quality was scored from 0 (higher quality) to 3 (lower quality). LC-OCT and RCM confidence level was scored from 0 (higher confidence level) to 3 (lower confidence level).

LC-OCT				
**For all lesions** **(*n* = 84)**	**Quality = 0** **(*n* = 4)**	**Quality = 1** **(*n* = 58)**	**Quality = 2** **(*n* = 18)**	**Quality = 3** **(*n* = 4)**
Average confidence level	0.8	1.2	1.4	2.3
For lesions also imaged with RCM (*n* = 36)	Quality = 0 (*n* = 1)	Quality = 1 (*n* = 27)	Quality = 2 (*n* = 7)	Quality = 3 (*n* = 1)
Average confidence level	1	1.1	1.3	3
**RCM**				
***n* = 36**	**Quality = 0** **(*n* = 0)**	**Quality = 1** **(*n* = 26)**	**Quality = 2** **(*n* = 9)**	**Quality = 3** **(*n* = 1)**
Average confidence level	-	1.1	1.4	2

**Table 2:** Comparison of image quality and confidence level between LC-OCT and RCM.

	LC-OCT	RCM
Image quality	*n* (%)	*n* (%)
Score 0	1 (2.8)	0 (0)
Score 1	27 (75.0)	26 (72.2)
Score 2	7 (19.4)	9 (25.0)
Score 3	1 (2.8)	1 (2.8)
Confidence level	*n* (%)	*n* (%)
Score 0	0 (0)	0 (0)
Score 1	31 (86.1)	28 (77.8)
Score 2	4 (11.1)	8 (22.2)
Score 3	1 (2.8)	0 (0)

**Table 3:** LC-OCT performances for diagnosing melanomas vs. nevi (dysplastic or not) compared to histology.

All Lesions		Histology			
		Melanoma	Nevus	Other	Total
LC-OCT	Melanoma	26	0	0	26
	Nevus	2	55	0	57
	Other	0	0	1	1
	Total	28	55	1	84

**Table 4:** LC-OCT performances for diagnosing melanomas vs. nevi vs. dysplastic nevi compared to histology.

All Lesions		Histology				
		Nevus	Melanoma	Dysplastic Nevus	Others	Total
LC-OCT	Nevus	40	1	8	0	49
	Melanoma	0	26	0	0	26
	Dysplastic nevus	2	1	5	0	8
	Others	0	0	0	1	1
	Total	42	28	13	1	84

**Table 5:** LC-OCT performances for diagnosing melanomas vs. nevi (dysplastic or not).

	*n*	Global Accuracy	Nevus (*n* = 42)			Dysplastic Nevus (*n* = 13)			Melanoma (*n* = 28)		
			Accuracy	Sensitivity	Specificity	Accuracy	Sensitivity	Specificity	Accuracy	Sensitivity	Specificity
**LC-OCT**	83	86%	87%	95%	78%	87%	38%	96%	98%	93%	100%

**Table 6:** LC-OCT and RCM performances for diagnosing melanomas vs. nevi (dysplastic or not) compared to histology.

*n* = 36		Histology			
		Melanoma	Nevus	Other	Total
LC-OCT	Melanoma	13	0	0	13
	Nevus	1	21	0	22
	Other	0	0	1	1
	Total	14	21	1	36
RCM	Melanoma	13	1	1	15
	Nevus	1	20	0	21
	Other	0	0	0	0
	Total	14	21	1	36

**Table 7:** LC-OCT and RCM performances for diagnosing melanomas vs. nevi vs. dysplastic nevi compared to histology.

*n* = 36		Histology				
		Nevus	Melanoma	Dysplastic Nevus	Others	Total
LC-OCT	Nevus	14	1	4	0	19
	Melanoma	0	13	0	0	13
	Dysplastic nevus	0	0	3	0	3
	Others	0	0	0	1	1
	Total	14	14	7	1	36
RCM	Nevus	13	0	3	0	16
	Melanoma	0	13	1	1	15
	Dysplastic nevus	1	1	3	0	5
	Others	0	0	0	0	0
	Total	14	14	7	1	36

**Table 8:** LC-OCT and RCM performances for diagnosing melanomas vs. nevi vs. dysplastic nevi.

	*n*	Global Accuracy	Nevus (*n* = 14)			Dysplastic Nevus (*n* = 7)			Melanoma (*n* = 14)		
			Accuracy	Sensitivity	Specificity	Accuracy	Sensitivity	Specificity	Accuracy	Sensitivity	Specificity
**LC-OCT**	35	86%	86%	100%	76%	89%	43%	100%	97%	93%	100%
**RCM**	35	83%	89%	93%	86%	83%	43%	93%	94%	93%	95%

**Table 9:** LC-OCT and RCM key criteria that were more useful in discriminating a melanoma from a nevus (dysplastic or not).

LC-OCT Parameters	OR (Univariate)	*p* Value	OR (Multivariate)	*p* Value
**Horizontal parameters**				
Irregular honeycombed pattern	43.64 (10.80–299.67)	<0.001	18.03 (1.50–547.32)	0.039
Pagetoid spread with atypical melanocytes in basal/suprabasal layers	41.21 (11.28–206.45)	<0.001	16.56 (1.43–435.95)	0.037
Edged papillae	0.22 (0.08–0.59)	0.003	0.43 (0.02–9.05)	0.57
Basal nests	0.23 (0.07–0.63)	0.007	0.51 (0.02–12.06)	0.67
Nests in the upper dermis	0.21 (0.06–0.61)	0.007	0.23 (0.01–3.80)	0.35
Irregular bright cells/sheets of cells in the upper dermis	5.75 (1.77–20.94)	0.005	0.51 (0.02–10.73)	0.67
**Vertical parameters**				
Pagetoid spread of bright cells in suprabasal/basal layers	20.36 (6.44–74.84)	<0.001	4.74 (0.35–94.88)	0.25
Junctional nests	0.31 (0.11–0.83)	0.02	1.85 (0.06–103.11)	0.73
Disturbed DEJ	10.83 (3.73–35.72)	<0.001	4.85 (0.26-144.46)	0.31
Dermal nests	0.21 (0.07–0.58)	0.004	0.08 (0.00-1.29)	0.12
Sheets of atypical bright cells	26.47 (4.50-506.78)	0.003	0.69 (0.02-29.63)	0.83
**RCM parameters**	**OR (univariate)**	***p* value**	**OR (multivariate)**	***p* value**
Irregular honeycombed pattern	69.67 (9.13–1553.6)	<0.001	123.91 (2.84–19309)	0.030
Pagetoid spread with atypical melanocytes in suprabasal layers	34.00 (5.91–315.56)	<0.001	0.76 (0.01–23.85)	0.88
Edged papillae	0.08 (0.01–0.44)	0.007	0.05 (0.00–0.76)	0.053

## Data Availability

Fully anonymized data are available on request.

## Supplementary Materials

The following are available online at https://www.mdpi.com/article/10.3390/cancers14051140/s1: Table S1: Most common LC-OCT parameters for melanocytic lesions; Table S2: LC-OCT key criteria more useful in discriminating a melanoma from a nevus (dysplastic or not); Table S3: Most common RCM parameters for melanocytic lesions; Table S4: RCM key criteria more useful in discriminating a melanoma from a nevus (dysplastic or not); Table S5: Analysis of the false negative cases.
