# Supplementary material for: Line-Field Confocal Optical Coherence Tomography: A New Tool for the Differentiation between Nevi and Melanomas?
Source: Cancers (Basel). 2022 Feb 23;14(5):1140. doi: 10.3390/cancers14051140 (PMC8909859; doi:10.3390/cancers14051140)
Supplement: Supplementary file 1 [file cancers-14-01140-s001.zip › cancers-1597647-supplementary.pdf]

Table S1. Most common LC-OCT parameters for melanocytic lesions.

N=77	Non-dysplastic nevus (N=39)	Dysplastic nevus (N=12)	All nevus (N=51)	Melanoma (N=26)
**LC-OCT horizontal parameters**				
Regular honeycombed pattern	33 (85)	8 (67)	41 (80)	2 (8)
Irregular honeycombed pattern	6 (15)	5 (42)	11 (22)	24 (92)
Pagetoid spread with atypical melanocytes in basal suprabasal layers	4 (10)	4 (33)	8 (16)	23 (89)
Edged papillae	24 (62)	12 (100)	36 (71)	9 (35)
Non-edged papillae	15 (39)	7 (58)	22 (43)	16 (62)
Basal hyperpigmentation	25 (64)	11 (62)	36 (71)	14 (54)
Basal nests	20 (51)	9 (75)	29 (57)	6 (23)
Nests in the upper dermis	20 (51)	7 (58)	27 (53)	5 (19)
Irregular bright cells in the upper dermis	4 (10)	1 (8)	5 (10)	10 (39)
**LC-OCT vertical parameters**				
Pagetoid spread of bright cells in suprabasal/basal layers	4 (10)	2 (17)	6 (12)	19 (73)
Basal hyperpigmentation	24 (62)	11 (92)	35 (69)	14 (54)
Junctional nests	22 (56)	10 (83)	32 (63)	9 (35)
Well-defined DEJ	28 (72)	10 (83)	38 (75)	10 (39)
Disturbed DEJ	10 (26)	2 (17)	12 (24)	20 (77)
Reticular acanthosis	16 (41)	6 (50)	22 (43)	6 (23)
Atrophy	9 (23)	1 (8)	10 (20)	4 (15)
Dermal nests	25 (64)	5 (42)	30 (59)	6 (23)
Sheets of atypical bright cells	1 (3)	0 (0)	1 (2)	9 (35)

Table S2. LC-OCT key criteria more useful in discriminating a melanoma from a nevus (dysplastic or not).

Parameters	OR (univariate)	P value
**Horizontal parameters**		
Regular honeycombed pattern	0.02 (0.00-0.08)	<0.001
Irregular honeycombed pattern	43.64 (10.80-299.67)	<0.001
Pagetoid spread with atypical melanocytes in basal suprabasal layers	41.21 (11.28-206.45)	<0.001
Edged papillae	0.22 (0.08-0.59)	0.003
Non-edged papillae	2.11 (0.81-5.68)	0.13
Basal hyperpigmentation (bright keratinocytes/basal layer)	0.49 (0.18-1.30)	0.15
Basal nests	0.23 (0.07-0.63)	0.007
Nests in the upper dermis	0.21 (0.06-0.61)	0.007
Irregular bright cells/sheets of cells in the upper dermis	5.75 (1.77-20.94)	0.005
**Vertical parameters**		
Pagetoid spread of bright cells in suprabasal/basal layers	20.36 (6.44-74.84)	<0.001
Basal hyperpigmentation	0.53 (0.20-1.41)	0.21
Junctional nests	0.31 (0.11-0.83)	0.02
Well-defined DEJ	0.21 (0.08-0.58)	0.003
Disturbed DEJ	10.83 (3.73-35.72)	<0.001
Reticular acanthosis	0.40 (0.13-1.10)	0.09
Atrophy	0.75 (0.19-2.52)	0.65
Dermal nests	0.21 (0.07-0.58)	0.004
Sheets of atypical bright cells	26.47 (4.50-506.78)	0.003

Table S3. Most common RCM parameters for melanocytic lesions.

N=34	Non-dysplastic nevus (N=13)	Dysplastic nevus (N=7)	All nevus (N=20)	Melanoma (N=14)
**RCM parameters**				
Regular honeycombed pattern	13 (100)	6 (86)	19 (95)	2 (14)
Irregular honeycombed pattern	0 (0)	1 (14)	1 (5)	11 (79)
Pagetoid spread with atypical melanocytes in basal suprabasal layers	1 (8)	2 (29)	3 (15)	12 (86)
Edged papillae	11 (85)	7 (100)	18 (90)	6 (43)
Non-edged papillae	1 (8)	3 (43)	4 (20)	10 (71)
Basal hyperpigmentation	10 (77)	7 (100)	17 (85)	10 (71)
Basal nests	9 (69)	5 (71)	14 (70)	6 (43)
Nests in the upper dermis	7 (54)	3 (43)	10 (50)	3 (21)
Irregular bright cells in the upper dermis	0 (0)	0 (0)	0 (0)	7 (50)

Table S4. RCM key criteria more useful in discriminating a melanoma from a nevus (dysplastic or not).

Parameters	OR (univariate)	P value
Regular honeycombed pattern	0.01 (0.00-0.07)	<0.001
Irregular honeycombed pattern	69.67 (9.13-1553.6)	<0.001
Pagetoid spread with atypical melanocytes in suprabasal layers	34.00 (5.91-315.56)	<0.001
Edged papillae	0.08 (0.01-0.44)	0.007
Non-edged papillae	10.00 (2.21-56.29)	0.005
Basal hyperpigmentation (bright keratinocytes/basal layer)	0.44 (0.07-2.39)	0.34
Basal nests	0.32 (0.07-1.30)	0.12
Nests in the upper dermis	0.27 (0.05-1.19)	0.10
Irregular bright cells/sheets of cells in the upper dermis	330425122 (0- NA)	0.99

Table S5. Analysis of the false negative cases.

Patient	Irregular honey-combed pattern (horizontal)	Pagetoid spread with atypical melanocytes in basal suprabasal layers (horizontal)	Dermal nests	Disturbed DEJ (vertical)	Comments
MUC0222	1	0	0	0	Histo: in situ SSMM + dysplastic nevus LC-OCT quality = 1; LC-OCT confidence = 2
MUC0300	0	0	0	1	Histo: in situ SSMM LC-OCT quality = 3; LC-OCT confidence = 3
